# Effect of Hydration on Gluten-Free Breads Made with Hydroxypropyl Methylcellulose in Comparison with Psyllium and Xanthan Gum

**DOI:** 10.3390/foods9111548

**Published:** 2020-10-26

**Authors:** Mayara Belorio, Manuel Gómez

**Affiliations:** College of Agricultural Engineering, University of Valladolid, 34004 Palencia, Spain; pallares@iaf.uva.es

**Keywords:** gluten-free bread, hydration, hydroxypropyl methylcellulose, xanthan gum, psyllium

## Abstract

The use of hydrocolloids in gluten-free breads is a strategy to improve their quality and obtain products with acceptable structural and textural properties. Hydration level (HL) optimization is important to maximize the hydrocolloids effects on dough and bread quality. This study evaluated the optimum hydration level (OHL) for gluten-free breads prepared with different starch sources (rice flour or maize starch) and hydroxypropyl methylcellulose (HPMC) in comparison with psyllium husk fibre and xanthan gum. Breads with the same final volume and the corrected hydration (CH) were evaluated. The hydration is a key factor that influences the final characteristics of gluten-free breads. Breads made with HPMC had greater dependence on the HL, especially for preparations with maize starch. Psyllium had similar behaviour to xanthan with respect to specific volume and weight loss. Breads manufactured with maize starch and HPMC had low hardness due to their great specific volume. However, in breads made with rice flour, the combined decreased hydration and similar specific volume generated a harder bread with HPMC than the use of psyllium or xanthan. Breads made with HPMC presented higher specific volume than the other hydrocolloids, however combinations among these hydrocolloids could be evaluated to improve gluten-free breads quality.

## 1. Introduction

Gluten plays an important role in bread formulation. The gluten network is formed by wheat proteins that with correct hydration and mechanical work, form a cohesive, extensible and elastic dough, which is able to retain the gas formed during fermentation and baking [1]. To elaborate gluten-free breads, it is necessary to resort to starches and gluten-free flours, but it is also important to replace gluten with another ingredient. However, a functionally equivalent ingredient has not yet been found that allows the full replacement of gluten. The most often used ingredients for this purpose are hydrocolloids [2,3,4,5].

Hydroxypropyl methylcellulose (HPMC) and xanthan gum are the hydrocolloids most often used as gluten substitutes in gluten-free breads, while rice flour and maize starch are the starchy ingredients most often employed in these formulations, both in scientific articles and in commercial products [6,7]. In commercial products, the use of psyllium is also prominent. In fact, Román et al. [7] indicated that 16% of all evaluated breads included psyllium as the major gluten replacer and 34% incorporated it as a secondary replacer, mixed with another main hydrocolloid. Similarly, as other hydrocolloids, psyllium is a natural fibre with important hydration and gel-forming properties [8]. It is an arabinoxylan composed by different monosaccharides and, as other hydrocolloids, it has many hydroxyl groups in its structure which increase its capacity to bind water and generate viscous solutions [9,10]. The use of psyllium has some advantages because, besides being a natural product, it is responsible for health benefits such as the regulation of glucose in diabetic disease and decreased symptoms of constipation, diarrhoea, irritable bowel syndrome and others [11]. Psyllium and xanthan gum present similar rheological behaviours, as both are responsible for weak gelling properties [12]. Nevertheless, studies about the elaboration of gluten-free breads with psyllium are scarce, and this fibre has always been studied in mixtures with other hydrocolloids such as HPMC and xanthan gum [13,14,15] but never as a unique gluten replacer. 

Dough hydration in gluten-free bread is a fundamental aspect of final product quality. In general, it is known that the greater the hydration, the higher the specific volume of a bread, until a maximum point at which the weak structure of the dough promotes collapse during the fermentation or baking process [16,17]. However, these studies were based on doughs elaborated with HPMC, and there is little or no information about the effect of hydration on doughs made with other hydrocolloids. Generally, previous scientific studies applied the same hydration levels, despite possible changes in the formulation, or they modified hydration based on pre-proofs which were not detailed. Some authors have attempted to correct hydration by performing rheological analysis (rheometer or farinographic) [18,19,20,21]. Similarly, Ren et al. [22] evaluated the effect of hydration in psyllium and methylcellulose breads using a response surface design. However, this system had an important limitation, due to the amount of each hydrocolloid used in the analysis. These quantities did not cover a wide range of concentrations and they were the same for all hydrocolloids, so the optimal point is usually difficult to find for each case. Nevertheless, Sahagún and Gómez [23] proved that distinct gluten-free formulations achieve a maximum specific volume with differences in both hydration levels and rheological properties. Furthermore, hydration influences bread volume differently, depending on the formulation used.

Gluten-free breads prepared with the most used gluten-substitutes (HPMC or xanthan gum) were compared with breads made with psyllium in doughs with rice flour or maize starch. Therefore, the objective of this study was to evaluate the different effect of using psyllium as a gluten replacer in gluten-free breadmaking. The influence of these hydrocolloids was evaluated on dough hydration and on the specific volume of the final breads. For each case, breads with the highest specific volume were analysed in terms of crust colour and texture (hardness, springiness, cohesiveness, chewiness and resilience).

## 2. Materials and Methods 

### 2.1. Bread Ingredients

Gluten-free breads were made with rice flour containing 7.54 g/100 g of protein (Molendum Ingredients SL, Zamora, Spain) or maize starch (Tereos, Syral Iberia SAU, Zaragoza, Spain). Other ingredients used were refined sunflower oil (Urzante, Navarra, Spain), sucrose (AB Azucarera Iberica, Valladolid, Spain), instant dry baker’s yeast (Dosu Maya Mayacilik A.S, Istanbul, Turkey), salt (Disal, Unión Salinera de España S.A, Madrid, Spain) and tap water. Gluten replacers were hydroxypropyl methylcellulose (HPMC) (Vivapur K4M, J. Rettenmaier and Söhne, Rosenberg, Germany), xanthan gum (Industrias Roko S.A., Llanera, Asturias, Spain) and psyllium husk fibre (psyllium P95) with 80% of total fibre (14% insoluble and 66% soluble, data provided by the supplier Rettenmaier Ibérica, Barcelona, Spain) and 3.42 of water holding capacity [8].

### 2.2. Gluten-Free Breadmaking

A gluten-free bread recipe was composed as follows (per 100 g flour or starch): 100 g of maize starch (MS) or rice flour (RF), 6 g of sunflower oil, 5 g of sugar, 3 g of yeast powder, 1.8 g of salt and 2 g of hydrocolloid (HPMC, xanthan gum or psyllium). The amount of water was defined according to topic 2.3.

All the ingredients were mixed by using a Kitchen Aid Professional mixer (Kitchen Aid, St. Joseph, MI, USA) with a dough hook (K45DH) at 58 rpm for 1 minute, except for the dry yeast and tap water. During this minute, the water was placed in a plastic container, the dry yeast was gently laid on top of the water and it was carefully mixed with the use of a glass rod to guarantee the hydration of the whole yeast. Subsequently, the hydrated yeast was mixed (90 rpm for 8 into the dough. Portions of bread dough (150 g) were placed into aluminium pans (127 × 98 × 33 mm) previously coated with sunflower oil. The dough was fermented in a proofing chamber at 30 °C and 80% relative humidity for 60 min. The fermented doughs were baked at 190 °C for 40 minutes. The aluminium pans were removed, and the bread was allowed to cool for 60 min and placed in plastic bags, which were closed properly and stored at 20 °C for 24 h until subsequent analysis. All studied formulations were produced in duplicate.

### 2.3. Defining the Optimum Hydration Level of Breads

The influence of hydration level was evaluated for each bread formulation considering the use of RF, MS and the different hydrocolloids (HPMC, xanthan gum and psyllium husk fibre), similarly to a study by Sahagún and Gómez [23]. Breads were made with formulations containing 70, 80, 90, 100, 110 and 120 g/100 g of water. Their specific volumes were obtained, and those with the maximum specific volume were considered to have the optimum hydration level (OHL). The volume of all breads was measured by using a Volscan Profiler 300 (Stable Microsystems, Surrey, UK), and the specific volume was calculated as the ratio between the final volume and weight of breads, 24 h after baking. Four loaves of bread were evaluated for each formulation.

To evaluate the physical characteristics of the breads, the OHL was chosen. The amount of dough to be used in the aluminium pans was recalculated for each formulation with the aim of obtaining the same final bread volume (680 mL). However, this volume exceeded the mould capacity, and it was observed that while some doughs could grow beyond the moulds, others overgrew it and exceeded the upper edge of the pan, causing the dough drop outside the mould without increasing the bread volume. In these cases, dough hydration was reduced to the percentage at which the bread could rise without the dough dropping outside the mould.

### 2.4. Physical Characteristics of Breads

The weight lost during baking was calculated as the difference between the bread and dough weights divided by the dough weight.

The texture of two central slices (20 mm thick) from two breads of each formulation was evaluated. A TPA (texture profile analysis) was performed by using a TA-XT2 texture analyser (Stable Micro Systems, Godalming, UK) with a cylindrical probe 25 mm in diameter. The probe penetrated 50% of the depth of each slice, with a trigger force of 5 g and a test speed of 1 mm/s. A delay of 10 s between the first and second compressions was applied. Hardness, springiness, cohesiveness and resilience were measured.

Crumb colour was measured by using a Minolta CM-508i spectrophotometer (Minolta Co., Ltd., Osaka, Japan) with D65 as the standard illuminant and a 2° standard observer. The results were expressed in the CIE L*a*b* colour space. Measurements were made on two central slices of two breads from each formulation (2 × 2 × 2).

### 2.5. Statistical Analysis

Analysis of variance (ANOVA) was performed with Statgraphics Centurion XVI software (Statpoint Technologies, Inc., Warrenton, VA, USA) to evaluate all the results obtained. The 95% confidence intervals were described by Fisher’s least significant differences (LSD) test.

## 3. Results

### 3.1. Optimum Hydration Level

**Figure 1:**
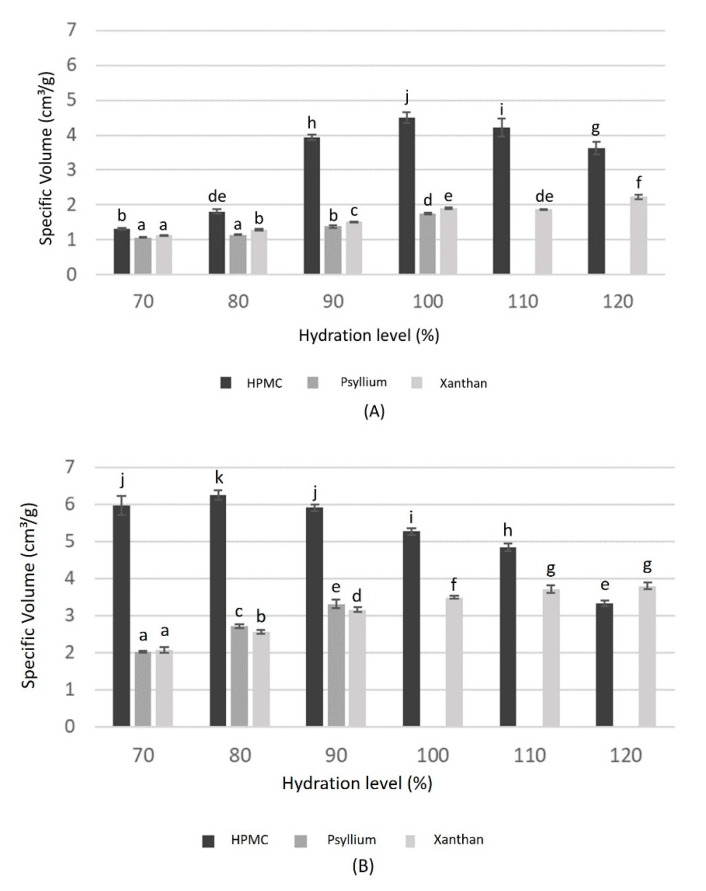
Variation of specific volume at different hydration levels for each gluten-free bread formulation and hydrocolloid: (**A**) rice flour (RF); (**B**) maize starch (MS). Same letters above bars means that there are no significant differences between those values (*p* < 0.05).

Specific volumes for each hydration level of gluten-free breads made with different hydrocolloids are shown in Figure 1. Among breads made with RF (Figure 1a), the specific volume of all breads increased with high hydration levels up to 100 g water/100 g flour. This increase was much larger in breads with HPMC than in those with psyllium husk fibre (PHF) or xanthan gum. In fact, breads with HPMC had a specific volume more than double of those obtained with the other hydrocolloids at 100% hydration, whereas at 70% hydration, they had similar specific volumes. The highest specific volume observed in breads elaborated with HPMC is in accordance with the results of Sabanis and Tzia [3]. Nevertheless, their study showed smaller differences than those found in this research, as breads prepared with HPMC were less hydrated than those formulated with xanthan, which reduced their volume. This is related to the water retention capacity of xanthan gum, which generates more viscous doughs. However, as shown in Figure 1, breads made with HPMC with a similar hydration of those made with xanthan gum, presented higher specific volume. The behaviour of HPMC is related to its capacity to form a thermo-reversible gel during baking, which increases the viscosity and establishes gas cell walls, providing high volume by preventing moisture loss [24]. From the 100% hydration level, the specific volume of breads with HPMC decreased, while the pattern for breads made with xanthan was a reduction at 110% and a slightly increase at 120%. However, at this hydration level the volume of the dough was reduced during fermentation, and a small increase was observed during baking. As the final volume of breads made with xanthan gum was not improved at 120% hydration, the OHL was defined as 100%, considering that at this level, the highest specific volume was obtained without the dough dropping during fermentation or baking. An increasing in the specific volume with increasing hydration up to a certain limit was also observed by Mancebo et al. [16] in breads elaborated with RF and by Sahagún and Gómez [23] in breads with MS. Both studies analysed breads with HPMC. Mancebo et al. [16] reported the specific volume of breads with optimum values of G^′^ and G^″^. Sahagún and Gómez [23] found that these rheological values depended on the bread formulation. Ziobro et al. [20] evaluated breads made with starches, guar gum and pectin, and they showed that there is a viscosity limit value at which bread volume decreases during baking. In fact, Mir et al. [2] affirmed that the internal viscosity of doughs should not be too low to avoid the release of bubbles during baking. Encina-Zelada et al. [25] also observed that the specific volume of breads made with xanthan gum or guar gum increased with increasing hydration. In this case, a relation with dough rheology was also mentioned, and it was shown that for high levels of xanthan it was necessary the addition of extra water.

In the case of PHF, doughs greater than 100% of hydration, although growing during fermentation, dropped over the edges of the moulds during baking. It is possible that the viscosity was reduced during the early stages of baking (before gelatinization) because of the increase in temperature, which promoted excessively liquid doughs with a weak structure that dropped over the edges of the mould. Thus, it was not possible to obtain properly baked rice bread containing 2% of PHF over 100% hydration (Figure 1). The specific volume of breads made with PHF was similar to those made with xanthan gum. This could be related to the rheological properties of psyllium husk fibre, which are very similar to those of xanthan gum [12].

Among breads made with MS, those with HPMC had the highest specific volume at the optimum hydration of 80% (Figure 1), and their volume gradually decreased with increasing hydration. This optimum is similar to the results obtained by Sahagún and Gómez [23] using a very similar formulation. Breads with xanthan gum presented an OHL at 110% hydration, because at 120% the volume of the dough decreased during fermentation and increased again during baking, but it was not larger than that obtained with 110%, and there was no significant difference between the two hydration levels. MS breads with PHF increased in specific volume up to 90% hydration; however, at this level, breads were completely hollow, which indicated that the dough structure was too weak and the interior matrix sunk during baking, while at the external surface, a thin crust was formed because of drying that occurred at the beginning of baking. As a result, it was not possible to measure these breads because of their weak structures. MS breads behaved similarly to RF breads, as formulations with HPMC at the OHL had nearly double the specific volume of those breads elaborated with psyllium husk fibre or xanthan gum; the differences between the latter two were small.

It is important to highlight that breads made with MS had a higher specific volume than those made with RF, considering all hydration levels and the different hydrocolloids used. All hydrocolloids (HPMC, PHF and xanthan gum) increased the final specific volume of breads by almost 50%, considering the maximum specific volume obtained for each of them. The highest specific volumes were previously found when using HPMC, comparing breads elaborated with MS to those with RF [15,26]. Martinez and Gómez [26] hypothesized that this different behaviour of RF and MS doughs could be attributed to a higher consistency of rice flour in respect to maize starch, probably due the presence of proteins. The authors also suggested another possible explanation, which is based on the presence of a protein layer that covers the starch granules of the flour, modifying the pasting behaviour and increasing the pasting temperature. With respect to the OHL, breads made with HPMC clearly had lower OHL in the presence of MS than in the presence of RF, but in the case of xanthan gum, this value was the same with MS and RF, considering that with RF the specific volume did not have significant difference when the hydration increased from 110% to 120%. In the case of PHF, it was not possible to compare values of OHL, since they were not determined by considering the maximum specific volume but because of structural problems discovered in the cases of high hydration levels. However, considering the use of psyllium husk fibre, the OHL was larger with MS (sinking of the internal structure) than RF (dough dropped outside of the mould during baking).

### 3.2. Gluten-Free Bread Properties

**Figure 2:**
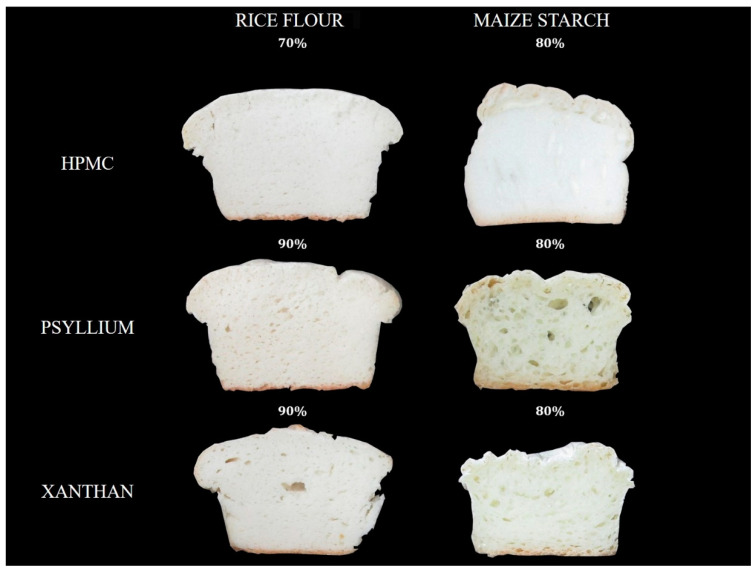
Crumb from maize starch or rice flour gluten-free breads and different hydrocolloids.

The initial idea was to produce breads with the same specific volume by changing the amount of water in the formulation, so this factor would not influence the study of the texture. It was also considered that the doughs could expand above the mould without overflowing and the hydration was reduced in the case it happened. However, the specific volume of breads made with maize starch had to be a little higher than those made with rice flour, since it was not possible to obtain such low specific volumes with MS. On the other hand, in breads made with maize starch and HPMC, it was not possible to achieve specific volumes as low as those obtained with psyllium husk fibre or xanthan gum, since reducing the hydration to a great extend generated excessively dry doughs that were difficult to handle. Table 1 shows the hydration levels used (corrected hydration, CH) and the specific volumes of breads elaborated with the CH. In the case of RF breads, it was necessary to use less hydration with the HPMC to obtain equal specific volumes of breads made with PHF and xanthan gum, according with the results obtained in the first part of this study. The CH of psyllium husk fibre and xanthan gum breads was slightly lower than the optimum found in the first part, since with the optimum hydration the doughs lost volume when they went over the edge of the mould. PHF and xanthan breads were also inferior to the optimum, due to the same problem found in RF breads, but somewhat superior to RF bread, as the doughs with these hydrations did not overflow. Regarding breads with HPMC, as it was not possible to achieve the specific volume, the hydration was corrected. In the cases in which it was not possible to equalize the specific volume, the amount of dough added in the moulds was modified to obtain breads with a similar final volume, as shown in Figure 2. Thus, all the breads had the same surface area, and this factor did not influence the weight loss after baking.

The weight loss during baking (Table 1) had no significant differences between breads made with RF. However, breads made with MS presented higher weight losses than those made with RF, and among these, those made with HPMC were the ones that presented the highest losses. In general, these weight losses are related to the volume of the loaves and the surface area, in a way that the greater is the volume, and therefore the biggest is the exchange surface, the greater will be the weight losses [16]. However, in this study, all breads had the same final volume and the same exchange surface area, so the changes in the loss of water during the baking process must be attributed to the different water absorption capacity of the ingredients used. This explains the absence of differences between breads made with PHF and xanthan, as the mixtures between starch and both ingredients have similar capacity to absorb water [8]. The higher weight loss in HPMC breads made with MS may be due to the smaller water holding capacity of this hydrocolloid [27]. The differences between starch and rice flour breads may be due to the lower water holding capacity of starches compared to flours [28], which may be related to the higher protein content of flours. In addition, a very compact structure, due to the lower specific volume of the RF breads, can also decrease the loss of water.

The results for breads texture (Table 2), show no significant differences in hardness between MS breads made with psyllium husk fibre or xanthan gum, but those made with HPMC have a considerably lower hardness. This may be due to the higher specific volume of these breads, since, in general, the higher is the specific volume, the lower is the hardness, as has been observed in previous studies with HPMC [3]. In fact, this relation between specific volume and hardness was indicated in other studies [16,26,29]. For the same reason, several studies found an increase in the hardness of gluten-free breads with the use of xanthan gum when compared with other hydrocolloids [30,31]. However, breads made with HPMC, despite of being softer, have been described as drier and with a crumblier texture [32]. Contrary to what has been observed for MS bread, in RF breads, where specific volumes were equalized, breads with HPMC presented much greater hardness than the others. This result is not observed when these products present a greater specific volume. The greater hardness of breads with HPMC found in this study may be attributed to the gels reverting to a weakly entangled form upon cooling, which increased crumb firmness after baking [29,33]. Nevertheless, despite the higher specific volume of breads elaborated with MS, RF breads made with PHF showed similar hardness and those with xanthan gum were less hard than MS breads. These differences can be explained by the distinct effect of xanthan gum on the pasting properties of starches, which includes the retrogradation phenomenon [28]. Thus, it seems that maize starch generates harder breads than rice flour. This is in accordance with the findings of Mancebo et al. [34], who reported that the texture of breads made with starch was harder than that of breads made with rice flour. RF breads made with xanthan and psyllium fiber husk showed higher springiness, cohesiveness and resilience in respect to RF-HPMC breads, possibly due to the same reason, regarding the changes in the HPMC gels after baking. On the contrary, in MS breads these changes were not found, as they were counteracted by the effect of the specific volume of the breads. As regards the PHF and xanthan breads, they showed almost similar values, and only slightly significant differences could be seen in MS breads, where PHF breads showed greater cohesiveness and resilience than xanthan breads.

Comparing the crust colour (Table 3), breads made with xanthan gum were darker (small values of L*) than breads with HPMC, and no significant differences were observed between breads made with HPMC and PHF. Neither were significant differences observed between RF breads elaborated with PHF or xanthan gum. Values of a* and b* had small significant differences, and no clear tendency was observed. However, breads made with xanthan gum had the largest values of a* in preparations containing RF and the smallest values of b* among those made with MS. Breads containing HPMC presented the highest values of a* and b* among preparations with MS. The crust colour of breads is related to the Maillard reaction, which occurs between amino acids and reducing sugars, as well as sugar caramelization [35]. Differences in sugar content and amino acids should not exist between breads elaborated with different hydrocolloids, since the other ingredients, which are the responsible for sugar and amino acid contents, did not change in the formulation. However, water activity can vary depending on the hydrocolloid and hydration of the doughs and this can affect Maillard reactions favouring the mobility of reactants [36]. In fact, Sabanis and Tzia [3] also found significant differences among the crust colours of breads made with distinct hydrocolloids.

## 4. Conclusions

In general, breads manufactured with HPMC and maize starch showed higher specific volumes than preparations with other hydrocolloids or rice flour. Nevertheless, the degree of hydration of the dough can change these results. The hydration effect is much more evident in breads prepared with HPMC than in those made with psyllium or xanthan gum. Thus, optimization of hydration is fundamental when different gluten-free breads are evaluated. After obtaining similar specific volume due to the corrected hydration, breads made with RF and HPMC were harder and less cohesive than breads made with psyllium or xanthan gum. It was not possible to obtain breads with the same specific volume because of great differences among the different hydrocolloids. Thus, breads made with HPMC and MS presented higher specific volume and lower hardness, but they presented a high weight loss during baking. Psyllium behaved similarly as xanthan gum, both with rice flour and maize starch. It is important to optimize the hydration for all gluten-free bread formulations made by using RF or MS with PHF, xanthan gum or HPMC. The optimum hydration allows to achieve high specific volumes, however in HPMC breads with RF and, especially, with MS it is possible to obtain specific volumes higher than breads with PHF or xanthan gum. Thought, it is necessary to study the combination between these hydrocolloids and starch sources to optimize the texture of final breads.

## Figures and Tables

**Table 1:** Optimum hydration level, specific volume and weight loss for each gluten-free bread and hydrocolloid used in the formulation.

Samples	CH (%)	Specific Volume (cm^3^/g)	Weight Loss (g/100g)
RF HPMC	70	1.33 ± 0.01 ^a^	9.33 ± 0.88 ^a^
RF Psyllium	90	1.44 ± 0.02 ^ab^	9.89 ± 0.18 ^a^
RF Xanthan	90	1.48 ± 0.03 ^b^	9.76 ± 0.18 ^a^
MS HPMC	80	7.58 ± 0.04 ^d^	28.20 ± 0.12 ^c^
MS Psyllium	80	2.37 ± 0.08 ^c^	16.60 ± 0.86 ^b^
MS Xanthan	80	2.25 ± 0.08 ^c^	17.50 ± 1.50 ^b^

Data are expressed as means ± Standard Deviation (SD) of duplicate assays. Values with the same letter in the same column do not present significant differences (*p* < 0.05). CH: corrected hydration. RF: rice flour. HPMC: hydroxypropyl methylcellulose. MS: maize starch.

**Table 2:** Texture parameters of gluten-free breads made with RF or MS for each hydrocolloid.

Samples	Hardness (N)	Springiness	Cohesiveness	Resilience
RF HPMC	42.44 ± 0.21 ^d^	0.796 ± 0.004 ^a^	0.656 ± 0.023 ^ab^	0.383 ± 0.009 ^a^
RF Psyllium	14.98 ± 0.60 ^c^	0.891 ± 0.025 ^b^	0.748 ± 0.037 ^c^	0.479 ± 0.041 ^bc^
RF Xanthan	9.04 ± 3.00 ^b^	0.922 ± 0.043 ^bc^	0.807 ± 0.024 ^c^	0.501 ± 0.013 ^bc^
MS HPMC	1.44 ± 0.12 ^a^	1.011 ± 0.023 ^d^	0.754 ± 0.030 ^c^	0.493 ± 0.034 ^bc^
MS Psyllium	19.51 ± 3.40 ^c^	0.974 ± 0.004 ^cd^	0.733 ± 0.037 ^bc^	0.550 ± 0.052 ^c^
MS Xanthan	19.58 ± 1.55 ^c^	0.964 ± 0.002 ^cd^	0.606 ± 0.037 ^a^	0.420 ± 0.047 ^ab^

Data are expressed as means ± SD of duplicate assays. Values with the same letter in the same column are not significantly different (*p* < 0.05). RF: rice flour. MS: maize starch.

**Table 3:** Crust colour parameters of gluten-free breads.

Samples	L*	a*	b*
RF HPMC	81.68 ± 3.05 ^c^	1.64 ± 0.25 ^bc^	17.15 ± 0.68 ^bc^
RF Psyllium	79.92 ± 4.67 ^bc^	1.23 ± 0.39 ^b^	15.36 ± 0.18 ^b^
RF Xanthan	75.05 ± 0.83 ^ab^	4.48 ± 0.08 ^d^	20.25 ± 1.22 ^c^
MS HPMC	82.09 ± 0.04 ^c^	2.64 ± 0.14 ^c^	19.32 ± 0.22 ^c^
MS Psyllium	86.20 ± 2.13 ^c^	-0.05 ± 1.07 ^a^	14.56 ± 2.64 ^b^
MS Xanthan	71.26 ± 1.92 ^a^	0.06 ± 0.09 ^a^	9.72 ± 1.93 ^a^

Data are expressed as means ± SD of duplicate assays. Values with the same letter in the same column are not significantly different (*p* < 0.05). RF: rice flour. MS: maize starch.
